# Newly Developed Mimetic Peptides for Angiotensin II Type 1 Receptor Attenuate Doxorubicin-Induced c-Jun N-Terminal Kinase Activation, a Marker of Pro-Apoptotic Stress Signaling

**DOI:** 10.3390/biomedicines14071464

**Published:** 2026-06-28

**Authors:** Yoshino Matsuo, Yasunori Suematsu, Shin-ichiro Miura

**Affiliations:** Department of Cardiology, Fukuoka University School of Medicine, Fukuoka 814-0180, Japan; yoshinoma@fukuoka-u.ac.jp (Y.M.); ysuematsu@fukuoka-u.ac.jp (Y.S.)

**Keywords:** angiotensin II mimetic peptides, doxorubicin, cardiotoxicity, c-Jun N-terminal kinase

## Abstract

**Objectives**: An ideal cardiotoxicity inhibitor targeting the angiotensin (Ang) II type 1 (AT_1_) receptor would be a β-arrestin-biased orthostatic ligand, which inhibits the G protein pathway and activates the β-arrestin pathway. Therefore, this study examined seven Ang II mimetic peptides (MP1–7), Ang A and TRV027 as potential β-arrestin-biased AT_1_ receptor ligands to prevent doxorubicin (Dox)-induced cardiotoxicity. **Methods**: Competition binding study, inositol phosphate (IP) production assay and extracellular signal-regulated kinase (ERK) 1/2 activation were performed using COS7 cells. Changes in phosphorylated Akt (Ser473), c-Jun N-terminal kinase (JNK) (Thr183/Tyr185), Bad (Ser112), Bcl-2 (Ser70), p53 (Ser46), active caspase-8 (Asp384) and active caspase-9 (Asp315) in cell lysates were measured using AT_1_ receptor-transfected H9C2 cells. **Results**: Binding assays showed Ang II and Ang A had the highest affinity, with MP2 and MP7 similar to TRV027. IP production was strong for Ang II and Ang A, minimal for MP1 and MP7, and no stimulation for MP2 and TRV027. Ang II and Ang A significantly activated ERK1/2 in this cell system. MP2 and MP7 in addition to TRV027 also significantly activated ERK1/2, whereas MP1 did not activate it. Dox-activated JNK and Bad, while Ang A, TRV027, MP2, and MP7 inhibited JNK activation without affecting Bad or Akt. **Conclusions**: MP2, which is a candidate biased ligand for the AT_1_ receptor and has similar amino acid sequence to TRV027, along with TRV027, attenuated Dox-induced JNK activation while avoiding excessive G protein-mediated activation. Interestingly, MP7, which showed minimal G protein-mediated activation with β-arrestin-mediated ERK activation, also attenuated Dox-induced JNK activation, a marker of pro-apoptotic stress signaling.

## 1. Introduction

The angiotensin II (Ang II) type 1 (AT_1_) receptor blockers (ARBs) are widely prescribed as antihypertensive and cardioprotective drugs [1,2,3,4]. Recently, cardio-oncology has attracted increasing attention, and doxorubicin-induced cancer therapy-related cardiac dysfunction (CTRCD), characterized by left ventricular dysfunction and heart failure, has become an important clinical problem in cancer patients receiving anthracycline therapy [5,6]. The renin–angiotensin–aldosterone system (RAAS) plays a central role in cardiovascular regulation and cardiac remodeling [2,3,4].

ARBs primarily inhibit the G protein-mediated signaling pathway of the AT_1_ receptor, thereby suppressing Ang II-induced vasoconstriction and exerting antihypertensive effects [2,7]. However, they may also inhibit β-arrestin-mediated signaling pathways that are thought to exert cardioprotective and anti-apoptotic effects [8]. Therefore, conventional ARBs may not be ideal for preventing anticancer drug-induced myocardial injury. Biased agonism is a pharmacological concept in which different ligands binding to the same G protein coupled receptor (GPCR) selectively activate distinct intracellular signaling pathways. In the case of the AT_1_ receptor, conventional Ang II stimulation activates Gq protein-dependent signaling, leading to phospholipase C activation, inositol trisphosphate (IP) production, protein kinase C activation, and mitogen-activated protein kinase signaling, including extracellular signal-regulated kinase (ERK)1/2 and c-Jun N-terminal kinase (JNK) pathways [7]. In contrast, β-arrestin-biased ligands activate β-arrestin-dependent signaling pathways while minimizing G-protein-mediated responses. β-arrestin not only contributes to receptor desensitization and internalization but also functions as a scaffold protein that mediates activation of signaling molecules [9]. Similar biased signaling mechanisms have also been reported in other GPCRs, including opioid receptor [10] and cannabinoid receptor [11].

Dox-induced cardiotoxicity clinically includes left ventricular dysfunction, cardiomyopathy, heart failure, arrhythmias, and, in severe cases, cardiovascular death. An ideal cardiotoxicity inhibitor targeting the AT_1_ receptor would be reasonable and a β-arrestin-biased orthosteric ligand that inhibits the G protein pathway while activating the β-arrestin pathway [12,13,14,15,16,17]. Biased agonism at G protein-coupled receptors has emerged as an important concept in drug development because it allows selective activation of beneficial signaling pathways while avoiding adverse signaling effects [12,13,14]. TRV027, a selective AT_1_ receptor biased ligand, has attracted attention in clinical trials for acute heart failure [12]. It selectively promotes β-arrestin recruitment and activates several kinase pathways, including ERK1/2 and Src [16]. In addition, TRV027-engaged AT_1_ receptor signaling has been reported to exert protective effects through molecular mechanisms distinct from those of conventional ARBs such as olmesartan [18]. TRV027 has unique vascular and hemodynamic effects. TRV027 reduces afterload while increasing cardiac performance and decreases cardiac contractility, cardiac output, and stroke volume [16]. In the phase IIb BLAST-AHF trial, TRV027 was well tolerated in patients with acute heart failure, although it did not significantly improve the primary clinical endpoint [12]. However, patients with higher baseline systolic blood pressure might derive greater hemodynamic benefit from TRV027 therapy [19]. However, despite promising pharmacological characteristics, the peptide agonist TRV027 has shown inconsistent efficacy in clinical trials and has not yet been approved for clinical use.

In this study, we designed seven newly developed Ang II mimetic peptides (MP1–7) and compared them with conventional peptide agonists to evaluate their potential as inhibitors of anticancer drug-induced myocardial injury through basic research.

## 2. Materials and Methods

### 2.1. Materials

We purchased the following reagents: Ang II and Ang A (Peptide Institute Inc., Osaka, Japan), ^125^I [Sar^1^, Ile^8^]Ang II (PerkinElmer Japan Co. Ltd., Tokyo, Japan), and doxorubicin (Dox). The Ang II MP1–7 were designed by substituting D-isomers of Ala and Ile as appropriate, while minimizing alterations to in the physicochemical properties of each of the eight amino acids in Ang II. These peptides were synthesized by Peptide Institute Inc. (Osaka, Japan) (Table 1).

### 2.2. Cell Cultures, Transfection, and Membrane Preparation

The synthetic rat AT_1_ receptor gene, cloned into the shuttle expression vector pMT-2, was used for expression as described previously [20,21]. COS7 cells and H9C2 cells (ATCC, Manassas, VA, USA; No. CRL-1651 and CRL-1446, respectively) were maintained under standard conditions. H9C2 cells are the original clonal cell line derived from embryonic rat heart tissue that exhibits many of the properties of skeletal muscle. Wild-type AT_1_ receptors were transiently transfected into the cells using Lipofectamine 2000 liposomal reagent (Roche Applied Science, Penzberg, Germany). Functional parameters were subsequently analyzed in the transfected cells. Cell membranes were prepared using either nitrogen Parr bomb disruption or freeze and thaw method.

### 2.3. Competition Binding Study

The dissociation constant (K_d_, nM) for receptor binding was determined using ^125^I-[Sar^1^, Ile^8^]Ang II in equilibrium binding assays. All experiments were performed under steady-state conditions to ensure accurate estimation of ligand–receptor affinity. Binding kinetics, including association and dissociation rates, were analyzed according to previously established methodologies [20,21]. These approaches allow precise characterization of receptor–ligand interactions and have been widely used in studies of Ang II receptor pharmacology.

### 2.4. IP Production Assay

Ang II peptide-induced IP production via the AT_1_ receptor was measured in transfected COS7 cells transiently expressing the AT_1_ receptor to evaluate Gq/PLC-mediated intracellular signaling. This pathway represents a key second messenger system in blood pressure regulation, in which IP_3_ generation leads to intracellular Ca^2+^ mobilization. In the present assay, total accumulated IPs (including IP_1_, IP_2_, and IP_3_) were quantified as an index of PLC activity. COS7 cells were transiently transfected with the AT_1_ receptor as described above and plated at appropriate density prior to assay. Cells were labeled with [^3^H]-myo-inositol for 24 h at 37 °C to inhibit inositol monophosphatase and thereby prevent degradation of accumulated IPs. After labeling, cells were washed and preincubated with assay buffer containing 10 mM LiCl for 20 min to inhibit inositol monophosphatase and prevent degradation of accumulated IPs. Cells were subsequently stimulated with Ang II peptides (1 μM) for 30 min at 37 °C. The reaction was terminated by rapid removal of the medium, and total soluble IPs were extracted using a perchloric acid extraction method. The extracts were processed, and radioactivity corresponding to IP accumulation was quantified by liquid scintillation counting. Nonspecific background signals were determined in unstimulated controls and subtracted from total values. All measurements were performed in duplicate or triplicate, and experiments were independently repeated at least three times to ensure reproducibility. All experimental procedures were performed in accordance with previously established methods [20,21], which are widely used in GPCR signaling studies. The methodological framework is consistent with established approaches in cardiovascular and renin–angiotensin–aldosterone system (RAAS) research, supporting the validity, reproducibility, and translational relevance of the findings [7,20,21].

### 2.5. Immunoblotting of ERK 1/2 Activation

ERK1/2 activation was evaluated in COS7 cells overexpressing AT_1_ receptor. Cells were serum-starved for 18 h in DMEM containing 0.1% serum to minimize basal ERK activity prior to stimulation. Cells were then stimulated with Ang II or its mimetic peptides (0.1 μM) for 10 min at 37 °C, a condition previously validated to induce robust ERK1/2 phosphorylation via AT_1_ receptor activation. Following stimulation, the culture medium was rapidly aspirated, and cells were washed with ice-cold phosphate-buffered saline to terminate signaling. Cells were immediately frozen in liquid nitrogen to preserve phosphorylation states. Samples were subsequently thawed, scraped, and homogenized in lysis buffer containing protease and phosphatase inhibitors. Protein concentrations were determined using the bicinchoninic acid method. Equal amounts of protein were separated by sodium dodecyl sulfate-polyacrylamide gel electrophoresis and transferred to a Hybond nitrocellulose membrane. Membranes were blocked with 10% bovine serum albumin in Tris-buffered saline (50 mM Tris-HCl, pH 7.6; 150 mM NaCl) for 1 h at room temperature and incubated overnight at 4 °C with antibodies against total ERK (#9102, Cell Signaling, Danvers, MA, USA) or phosphor (p)-ERK (#9106, Cell Signaling, Danvers, MA, USA). Horseradish peroxidase-conjugated secondary antibodies (Bio-Rad Laboratories Inc., Hercules, CA, USA) were then applied, and signals were detected using an enhanced chemiluminescence system (Amersham, Buckinghamshire, UK). Band intensities were quantified by digital image analysis using ImageJ (National Institutes of Health, Bethesda, MD, USA). The pERK1/2 levels were normalized to total ERK1/2, and results were expressed as fold change relative to unstimulated controls. All experiments were performed in at least duplicate and independently repeated to ensure reproducibility. These procedures were conducted in accordance with previously established methods [7,20,21], which are also widely used in GPCR signaling studies.

### 2.6. Measurement of Apoptotic Factors

Changes in phosphorylated Akt (Ser473), JNK (Thr183/Tyr185), Bad (Ser112), Bcl-2 (Ser70), p53 (Ser46), active caspase-8 (Asp384) and active caspase-9 (Asp315) in cell lysates of AT_1_ receptor in the transfected H9C2 cells were measured using MILLIPLEX^®^ Early Phase Apoptosis 7-plex Signaling kit (Merck, Millipore and Sigma-Aldrich, Darmstadt, Germany). Cell lysates were diluted, wells preconditioned, and beads along with samples or controls were added. Plates were incubated overnight. After magnetic separation and two washes, detection antibody was added for 1 h, followed by streptavidin-phycoerythrin and amplification buffer. Beads were then resuspended in assay buffer and analyzed using a calibrated Luminex^®^ system (Thermo Fisher Scientific K.K., Tokyo, Japan).

### 2.7. Statistical Analysis

All data are expressed as the mean ± standard deviation. Each experiment was performed in four or more independent determinations. Differences among measured values were evaluated by analysis of variance, followed by Fisher’s PLSD method (StatView-J 5.0, SAS Institute Japan Ltd., Tokyo, Japan). A *p*-value of <0.05 was considered statistically significant.

## 3. Results

### 3.1. The K_d_ Values of Ang II and Its Mimetic Peptides for the AT_1_ Receptor

The native peptide Ang II exhibited the highest binding affinity for the AT_1_ receptor, with a K_d_ value of 0.76 ± 0.22 nM (Table 1). The K_d_ value of Ang A (1.3 ± 0.6 nM) was nearly equivalent to that of Ang II. The K_d_ values of MP2 and MP7 were comparable to that of the biased ligand TRV027, whereas MP1 showed slightly reduced binding. In contrast, MP3 demonstrated markedly poor binding to the AT_1_ receptor, and MP4, MP5, and MP6 showed negligible binding, with measurements exceeding the assay’s limit. Based on these results, MP1, MP2, and MP7 were selected for subsequent experiments.

### 3.2. IP Production Using Ang II and Its Mimetic Peptides

**Figure 1:**
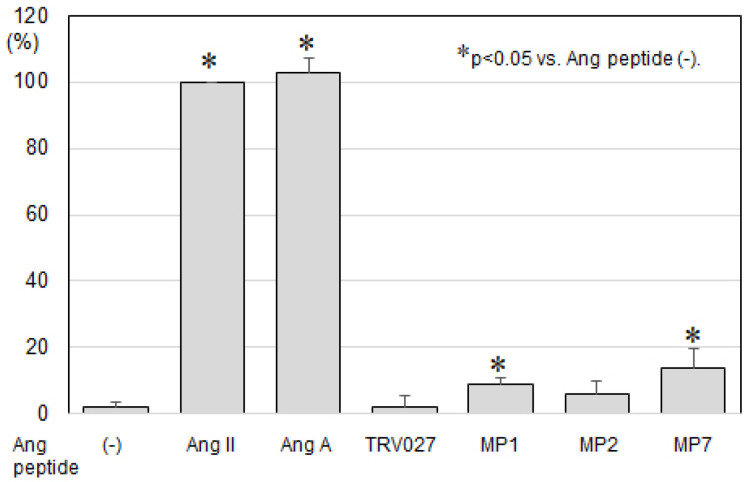
Measurements of inositol phosphate (IP) production using 0.1 μM of various Ang II and its mimetic peptides (n = 4 independent experiments, each measured in duplicate).

Next, we evaluated IP production as an index of G protein-dependent intracellular signaling mediated by the AT_1_ receptor (Figure 1). Ang II and Ang A elicited robust IP production, achieving comparable maximal responses, indicating full agonistic activity toward the AT_1_ receptor. In contrast, TRV027 and MP2 did not induce a significant increase in IP production compared with baseline levels, suggesting minimal activation of Gq-mediated signaling pathways.

### 3.3. Levels of ERK Activities Using Ang II Ant Its Mimetic Peptides

**Figure 2:**
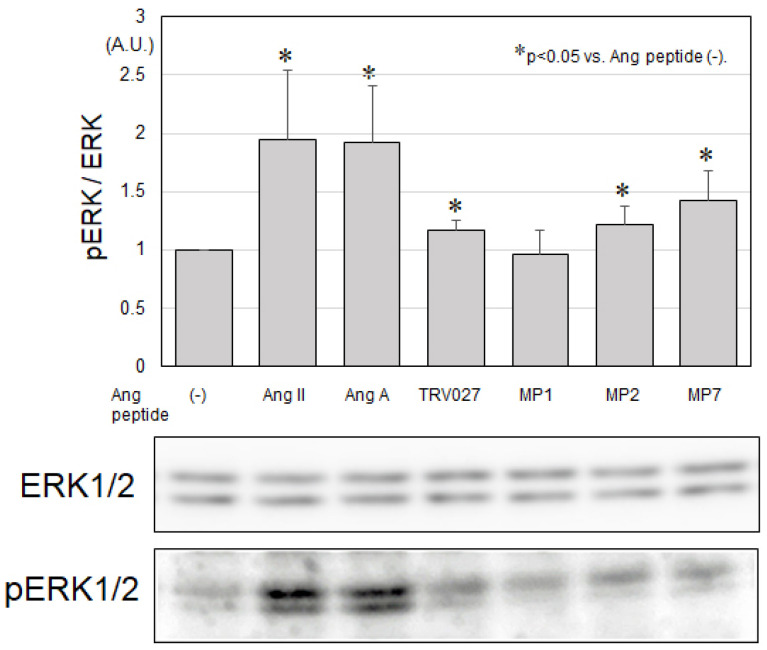
Levels of ERK activities using 0.1 μM of various Ang II and its mimetic peptides (n = 5 independent experiments, each measured in duplicate).

We next assessed ERK1/2 activation as a downstream signaling response to stimulation with Ang II and its mimetic peptides (Figure 2). Ang II and Ang A significantly increased ERK1/2 phosphorylation levels in this cell system, indicating robust activation of mitogen-activated protein kinase-dependent signaling pathways. In addition, TRV027, MP2, and MP7 also significantly enhanced ERK1/2 activity compared with baseline, suggesting that these ligands retain the ability to activate ERK signaling despite their differential effects on G protein-mediated pathways. In contrast, MP1 did not induce a significant increase in ERK1/2 activation, indicating limited involvement in this signaling cascade.

### 3.4. Determination of Phosphorylated JNK, Bad and Akt

**Figure 3:**
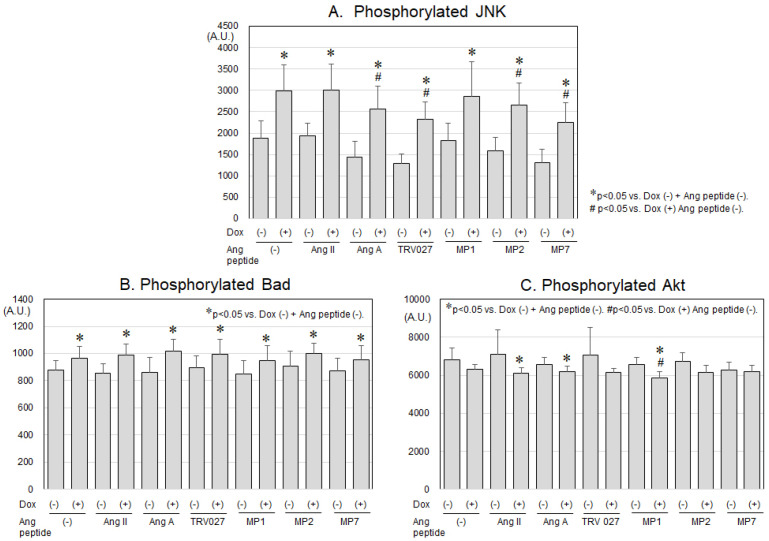
Measurements of phosphorylated JNK (**A**), Bad (**B**) and Akt (**C**) using 0.1 μM of various Ang II and its mimetic peptides under the presence or absence of 0.5 μM of Dox (n = 6 independent experiments, each measured in triplicate).

Changes in the phosphorylation levels of Akt, JNK, and Bad, as well as apoptosis-related proteins including Bcl-2, p53, active caspase-8, and active caspase-9, were assessed using the MILLIPLEX^®^ kit (Figure 3A–C). These analyses were performed to evaluate signaling pathways involved in Dox-induced JNK signaling. Dox treatment significantly increased the phosphorylation of JNK and Bad, indicating activation of pro-apoptotic signaling pathways. The Dox-induced activation of JNK was modestly attenuated by Ang A, TRV027, MP2, and MP7, suggesting a modulatory effect of these ligands on stress signaling. In contrast, Bad phosphorylation was not inhibited by any of the Ang peptides. Dox also reduced Akt phosphorylation, consistent with suppression of pro-survival signaling, and this effect was not reversed by any of the Ang peptides. In addition, Dox did not significantly alter the levels of Bcl-2, p53, active caspase-8, or active caspase-9.

## 4. Discussion

**Figure 4:**
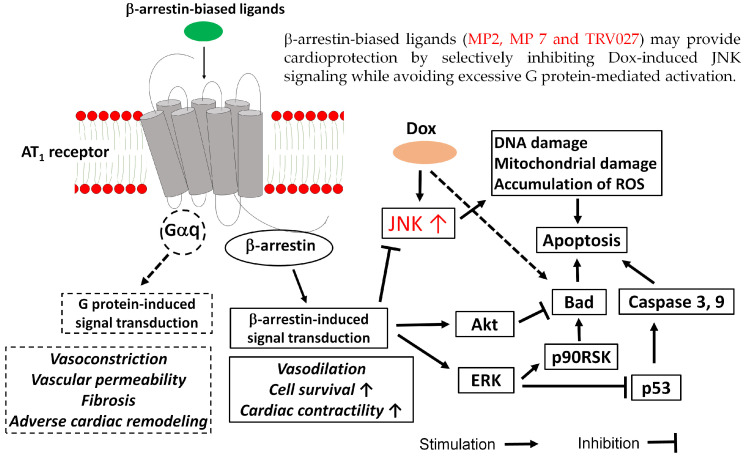
β-arrestin-biased orthosteric ligand and cardioprotection. MP, mimetic peptide; AT_1_, angiotensin II type 1; Dox, doxorubicin; JNK, c-Jun N-terminal kinase; ERK, extracellular signal-regulated kinase; ROS, reactive oxygen species. Solid arrows indicate signaling pathways supported by the present findings, whereas dashed arrows indicate pathways that appear to be minimally involved. Solid boxes represent effects predicted from the present findings, while dashed boxes represent effects not predicted from the present findings.

This study suggested that MP2, a candidate biased ligand for the AT_1_ receptor, along with TRV027, may attenuate doxorubicin-induced JNK activation, a marker of pro-apoptotic stress signaling (Figure 4). On the other hand, because the amino acid sequence of MP2 is similar to that of TRV027, MP2 cannot be regarded as a newly developed compound. Interestingly, MP7, which showed minimal G protein-mediated activation with β-arrestin-mediated ERK activation, also attenuated Dox-induced JNK activation. Thus, MP7 differs from TRV027 in its amino acid sequence, including the absence of D-Ala, and can therefore be regarded as a newly developed compound.

In TRV027, substitution of D-Ala at position 8 of Ang II has been introduced to confer resistance to enzymatic degradation and to stabilize ligand activity. In the present study, we further explored the structural impact of D-Ala substitution by introducing it not only at position 8 but also at position 4 (MP4 and MP5). However, these modified peptides failed to exhibit measurable binding affinity to the AT_1_ receptor, suggesting that substitution at position 4 critically disrupts receptor–ligand interactions and compromises receptor recognition. These findings highlight the importance of positional specificity in amino acid substitution for maintaining AT_1_ receptor binding.

The mechanisms underlying Dox-related CTRCD remain incompletely understood. Accumulating evidence suggests that multiple molecular processes contribute to Dox-induced cardiotoxicity, including DNA damage, mitochondrial dysfunction [22,23], and excessive generation of reactive oxygen species (ROS) [24,25]. These cellular stresses lead to impaired mitochondrial integrity, activation of apoptotic signaling pathways, and ultimately cardiomyocyte death. The integration of these mechanisms results in progressive myocardial dysfunction and clinical manifestations of heart failure.

Several intracellular signaling pathways have been implicated in Dox-induced apoptosis, including the JNK, ERK and p38 mitogen-activated protein kinase pathways [26,27,28]. These pathways are activated in response to cellular stress and play important roles in regulating cardiomyocyte survival and apoptosis. Among them, the JNK pathway is a direct activator of mitochondrial damage machinery in rat cardiac ventricular myocytes [28] (Figure 4). In the present study, Dox significantly increased JNK activation, consistent with its role as a central stress-responsive pathway. This activation was attenuated by Ang A, TRV027, MP2, and MP7, suggesting that modulation of AT_1_ receptor signaling can suppress JNK-mediated apoptotic signaling. Taken together, these findings indicate that inhibition of JNK signaling may represent an important mechanism underlying the cardioprotective effects observed in this study.

As another signaling pathway, activation of Akt phosphorylates and inhibits pro-apoptotic factors such as Bad, a member of the Bcl-2 family (Figure 4). However, in the present experimental system, Akt activation or inactivation was not clearly observed, and the involvement of the Akt pathway was considered inconclusive. In addition, Dox activated Bad, suggesting induction of apoptotic signaling. However, Ang II peptides did not suppress Dox-induced Bad, indicating that pathways independent of the AT_1_ receptor may be involved.

Recent studies have also suggested that some cardiometabolic drugs may attenuate Dox-induced cardiotoxicity. For example, empagliflozin attenuates Dox-induced apoptosis by inhibiting phosphorylation of the JNK/STAT3 signaling pathway and its downstream pathways, including ROS generation and NAD^+^ metabolism [29]. Similarly, previous studies using H9C2 cells—the same cell line employed in the present study—demonstrated that inhibition of STAT3 counteracted the protective effects of dapagliflozin on Dox-induced apoptosis [30,31]. These findings support the importance of intracellular stress signaling pathways in the development of cardiotoxicity.

More broadly, GPCR signaling in the heart is highly complex, and the balance between G protein-dependent and β-arrestin-dependent signaling is increasingly recognized as an important determinant of therapeutic effects [8,32,33,34]. Therefore, the development of biased ligands targeting AT_1_ receptors may represent a promising strategy for cardioprotection.

However, this study has several limitations. First, because MP2 shares a similar amino acid sequence with TRV027, it remains unclear whether it would exert stronger cardioprotective effects than TRV027 in vivo. Second, although MP7 attenuated Dox-induced JNK activation, it cannot be considered a classical biased ligand because it showed minimal G protein-mediated activation with β-arrestin-mediated ERK activation. Third, although MP2 and MP7 suppressed Dox-induced JNK activation, no direct effects were observed on the caspase system or Bcl-2 signaling pathways, which are closely associated with apoptosis. Additional studies are required to further clarify these mechanisms. Fourth, MP2 and MP7 should be considered “candidate biased ligands” rather than definitively characterized biased agonists. Future studies incorporating detailed concentration–response analyses and formal bias factor calculations will be necessary to confirm their pharmacological profiles. Finally, a limitation of this study is the lack of direct viability or apoptosis assays. Therefore, the findings should be considered suggestive of cardioprotective potential rather than definitive proof.

## 5. Conclusions

MP2, which is a candidate biased ligand for the AT_1_ receptor and has similar amino acid sequence to TRV027, along with TRV027, attenuated JNK activation while avoiding excessive G protein-mediated activation. Interestingly, MP7, which showed minimal G protein-mediated activation with β-arrestin-mediated ERK activation, also attenuated doxorubicin-induced c-Jun N-terminal kinase activation, a marker of pro-apoptotic stress signaling. Additional investigations are needed to elucidate the detailed mechanisms involved.

## Figures and Tables

**Table 1:** The K_d_ (nM) values of AT_1_ receptor binding of Ang II and its MP.

Ang Peptides	Amino Acid Sequences	K_d_ (nM)
Ang II	Asp^1^-Arg^2^-Val^3^-Tyr^4^-Ile^5^-His^6^-Pro^7^-Phe^8^	0.76 ± 0.22
Ang A	Ala^1^-Arg^2^-Val^3^-Tyr^4^-Ile^5^-His^6^-Pro^7^-Phe^8^	1.3 ± 0.6
TRV027	Sar^1^-Arg^2^-Val^3^-Tyr^4^-Ile^5^-His^6^-Pro^7^-D-Ala^8^	7.8 ± 3.3
MP1	Thr^1^-Arg^2^-Leu^3^-Tyr^4^-Lys^5^-His^6^-Pro^7^-Ile^8^	54 ± 6
MP2	Sar^1^-Arg^2^-Val^3^-Phe^4^-Ile^5^-His^6^-Pro^7^-D-Ala^8^	13 ± 6
MP3	Sar^1^-Arg^2^-Val^3^-Phe^4^-Gln^5^-His^6^-Pro^7^-D-Ala^8^	2417 ± 733
MP4	Asp^1^-Arg^2^-D-Ala^3^-Tyr^4^-Ile^5^-His^6^-Pro^7^-D-Ala^8^	>10^4^
MP5	Asp^1^-Arg^2^-D-Ala^3^-Tyr^4^-Gln^5^-His^6^-Pro^7^-D-Ala^8^	>10^4^
MP6	Gly^1^-Arg^2^-Val^3^-Phe^4^-Gln^5^-His^6^-Pro^7^-D-Ala^8^	>10^4^
MP7	Thr^1^-Arg^2^-Leu^3^-Tyr^4^-Ile^5^-His^6^-Pro^7^-Ile^8^	4.4 ± 1.6

AT_1_, angiotensin (Ang) II type 1; MP, mimetic peptide.

## Data Availability

The data presented in this study are available from the corresponding author on reasonable request.

## Abbreviations

The following abbreviations are used in this manuscript:

Ang	angiotensin
ARBs	Ang II type I (AT_1_) receptor blockers
CTRCD	cancer therapy-related cardiac dysfunction
Dox	doxorubicin
ERK	extracellular signal-regulated kinase
IP	inositol phosphate
JNK	c-Jun N-terminal kinase
MP	mimetic peptide
ROS	reactive oxygen species
